# Diagnostic criteria of small sellar lesions with hyperprolactinemia: Prolactinoma or else

**DOI:** 10.3389/fendo.2022.901385

**Published:** 2022-09-06

**Authors:** Anna Cho, Greisa Vila, Wolfgang Marik, Sigrid Klotz, Stefan Wolfsberger, Alexander Micko

**Affiliations:** ^1^ Department of Neurosurgery, Medical University of Vienna, Vienna, Austria; ^2^ Clinical Division of Endocrinology and Metabolism, Department of Internal Medicine III, Medical University of Vienna, Vienna, Austria; ^3^ Division of Neuroradiology and Musculoskeletal Radiology, Department of Biomedical Imaging and Image-guided Therapy, Medical University Vienna, Vienna, Austria; ^4^ Division of Neuropathology and Neurochemistry, Department of Neurology, Medical University of Vienna, Vienna, Austria; ^5^ Department of Neurosurgery, Medical University of Graz, Graz, Austria

**Keywords:** pituitary adenoma, prolactinoma, hyperprolactinemia, prolactin-volume-ratio, PVR

## Abstract

**Objective:**

To evaluate the combined predictive value of MRI criteria with the prolactin-volume-ratio (PVR) to differentiate prolactinoma from non-prolactinoma, in small sellar lesions with hyperprolactinemia.

**Methods:**

Retrospective analysis of 55 patients with sellar lesions of ≤15 mm diameter on MRI and hyperprolactinemia of ≤150 ng/mL, surgically treated between 2003 and 2020 at the Medical University of Vienna, with a conclusive histopathological report. Serum prolactin levels, extent of pituitary stalk deviation, size and volume of the lesion were assessed. The PVR was calculated by dividing the preoperative prolactin level by tumor volume.

**Results:**

Our study population consisted of 39 patients (71%) with a prolactin-producing pituitary adenoma (group A), while 16 patients (29%) had another type of sellar lesion (group B). Patients in group A were significantly younger (p=0.012), had significantly higher prolactin levels at diagnosis (p<0.001) as well as smaller tumor volume (p=0.036) and lower degree of pituitary stalk deviation (p=0.009). The median PVR was significantly higher in group A (243 ng/mL per cm^3^) than in group B (83 ng/mL per cm^3^; p=0.002). Furthermore, the regression operating characteristics analysis revealed a PVR >100 ng/mL per cm^3^ to be predictive for distinguishing prolactin-producing lesions from other small sellar lesions.

**Conclusion:**

In patients with small sellar lesions, Prolactin-Volume-Ratios >100 represents a possible predictive marker for the diagnosis of prolactin-producing pituitary adenomas.

## Introduction

Despite advances in MR imaging, neurosurgeons are still challenged with the differential diagnosis of small sellar lesions associated with mild hyperprolactinemia: Prolactinoma or else? (1, 2)

For prolactinomas, a serum prolactin level of 150 ng/mL is generally considered a diagnostic threshold. In these cases, standard treatment is medical by dopamine agonists (DA), as they cause a rapid decline in serum PRL levels with concurrent reduction in adenoma size (3).

For other small sellar lesions, such as non-functioning pituitary adenomas, Rathke’s cleft cysts, etc., treatment may be surgical if these lesions are symptomatic or enlarging (3).

However, such non-prolactin-producing sellar lesions may mimic prolactinomas due to compression of the pituitary stalk, which decreases the delivery of dopamine, causing hyperprolactinemia due to the missing inhibition effect of dopamine on lactotroph cells (4).

Hence, diagnostic criteria to allocate patients with small sellar lesions and mild hyperprolactinemia to the correct treatment (medical or surgical) are required (1, 2).

Recently, the use of the Prolactin-Volume-Ratio (PVR), has been proposed as a helpful diagnostic tool within the entity of pituitary adenomas to distinguish prolactinomas from other adenomas, independent of tumor size and volume (5).

The aim of this study was to evaluate the predictive value of preoperative MRI criteria as well as of the PVR to differentiate prolactinoma from non-prolactinoma in small sellar lesions with hyperprolactinemia.

## Materials and methods

For this study, our database of endoscopic endonasal transsphenoidal interventions at the Department of Neurosurgery, Medical University of Vienna between 2003 and 2020, was scanned for cases with small sellar lesions and elevated PRL levels.

Inclusion criteria included age ≥ 18 years, maximal diameter ≤ 15 mm (not impinging the optic chiasm), preoperative hyperprolactinemia ≤ 150 ng/mL (6), and a conclusive histopathological report. Patients with known history of pregnancy, breastfeeding, untreated primary hypothyroidism, medication-induced hyperprolactinemia as well as with clinical and biochemical signs of acromegaly or Cushing’s Disease were excluded. A total of 55 of 766 (7%) patients were enrolled in this study.

The study complied with the Declaration of Helsinki and was approved by the institutional ethics committee of the Medical University of Vienna (EK 2201/2020).

### Radiological, endocrine, and histopathological evaluation

Radiological parameters: In all patients, T1-weighted contrast-enhanced (CE) isovoxel MR images were available for evaluation. We assessed size and volume of the lesion, as well as the extent of pituitary stalk compression and deviation as preoperative radiological criteria.

Tumor size was measured as the maximum diameter of the lesion. Tumor volume was semi-automated calculated by tumor segmentation on radiological software (IMPAX EE R20 XIX, Agfa HealthCare N.V.). To evaluate if manual volume assessment on standard diagnostic coronal and sagittal T1-weighted CE MR images provides a possible alternative, we also calculated the volume by the approximation formula (width x height x length/2) on such image planes. The median tumor volume did not show any significant differences between both methods (p=0.106).

Involvement of the pituitary stalk was classified as compression or deviation. Stalk compression was defined as decreased length between optic chiasm and level of the sellar diaphragm. Stalk deviation was defined as the deviation angle of the pituitary stalk from the midline on coronal CE T1-weighted MRI (7).

Endocrine parameters: Prolactin level at diagnosis of the small sellar lesion, before the intake of dopamine agonists, was assessed in all patients. To assess remission rates, postoperative prolactin levels for prolactin-producing adenomas and radiological reports for other sellar lesions were additionally evaluated.

The Prolactin-Volume-Ratio was calculated by dividing the maximum preoperative prolactin level (ng/mL) by the semi-automated calculated tumor volume (cm^3^), as described previously (5).

Histopathological analysis was re-assessed according to the criteria of the WHO 2017 classification of tumors of endocrine organs (8). Depending on their histopathological classifications, our study patients were separated into two groups. Group A consisted of prolactin-producing adenomas (lactotroph and mammosomatotroph), while group B consisted of all other sellar lesions.

### Statistical analysis

Continuous variables were presented as median and interquartile ranges (IQR) and categorical variables as counts and percentages. The Chi-square and Mann-Whitney U tests were performed as appropriate for comparisons between the patient groups. Receiver Operating Characteristics (ROC) analyses were performed to identify the appropriate cutoff values of diagnostic potential of the Prolactin-Volume-Ratio. For all tests, p-values <0.05 were considered to be statistically significant.

Statistical analyses were carried out with IBM SPSS Statistics for Windows (Version 24 Armonk, NY: IBM Corp.).

## Results

### Patient characteristics

In our study population, the median age was 41 years (IQR 29 - 47) and the majority of patients were female (43/55, 78%). While 39 (71%) patients had a prolactin-producing pituitary adenoma (group A), 16 (29%) patients had a sellar lesion other than prolactinoma (group B). Pathologies of group B included other pituitary adenomas (7/16, 44%): 5 null cell adenomas, 2 gonadotroph adenomas; Rathke’s cleft cysts (7/16, 44%), hypophysitis (1/16, 6%) and intrasellar meningioma (1/16, 6%).

Primary symptoms leading to consultation were oligomenorrhea/amenorrhea in 21/55 (38%), incidental finding (13/55, 24%), headache (9/55, 16%), galactorrhea (6/55, 11%), erectile dysfunction/decreased libido (3/55, 5.5%) and weight gain (3/55, 5.5%)

### Comparison of prolactin-producing (group A) and non-prolactin-producing (group B) lesions

Patients in group A were significantly younger (37, IQR 29 - 44) than in group B (50, IQR 41-51; p=0.012). Sex distribution did not significantly differ within both groups (females within group A: 28/39, 72% vs. females within group B: 15/16, 94%; p=0.073).

For a detailed overview of clinical and radiological differences between patients with and without prolactin-producing small sellar lesions refer to **Table 1**.

**Table 1:** Baseline characteristics.

	Group A ( n = 39 )	Group B ( n = 16 )	p-values
**Age,** median (IQR)	37 (29 – 44)	50 (41 - 51)	**0.012**
**Female sex,** n (%)	15 (94%)	28 (72%)	0.073
**Preoperative prolactin level**, ng/mL, *median (IQR)*	90 (69 - 120)	53 (41 - 71)	**<0.001**
**Volume**, ccm^3^, *median (IQR)*	0.36 (0.21 - 0.50)	0.66 (0.28 - 0.97)	**0.036**
**Angle of pituitary stalk deviation**, °, *median (IQR)*	6 (0 - 13)	14 (11 - 27)	**0.009**
**Prolactin-Volume-Ratio,** (ng/mL)/ccm^3^, *median (IQR)*	243 (148 - 418)	83 (52 - 247)	**0.002**

IQR, interquartile range; PVR, Prolactin-Volume-Ratio.

Detailed overview of baseline characteristics for patients with prolactin-producing pituitary adenomas (group A) and patients with other kinds of sellar lesions (group B). Bold values indicate age = years; angle of pituitary stalk deviation = degree.

**Figure 1:**
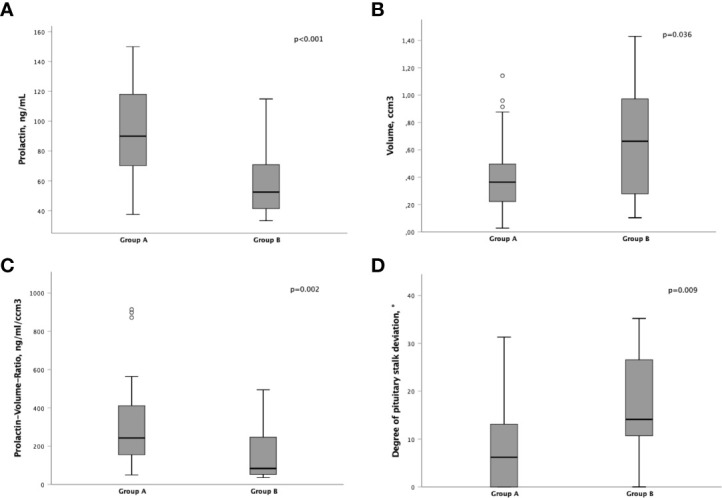
Differences of pre-operative parameters between group A and B,**(A)** Prolactin levels (ng/mL). **(B)** Tumor volume (ccm^3^). **(C)** Prolactin-Volume-Ratio (PVR; ng/mL/ccm^3^). **(D)** Degree of pituitary stalk deviation (°).

Patients in group A had significantly higher prolactin levels at diagnosis (90 ng/mL, IQR 69 - 120) than in group B (53 ng/mL, IQR 41 - 71; p<0.001; **Figure 1A**). Patients with a PRL level > 120 ng/mL were only found in group A.

Furthermore, the lesion volume was significantly smaller in group A (0.36 cm^3^, IQR 0.21 - 0.50 vs. 0.66 cm^3^, IQR 0.28 - 0.97; p=0.036; **Figure 1B**).

**Figure 2:**
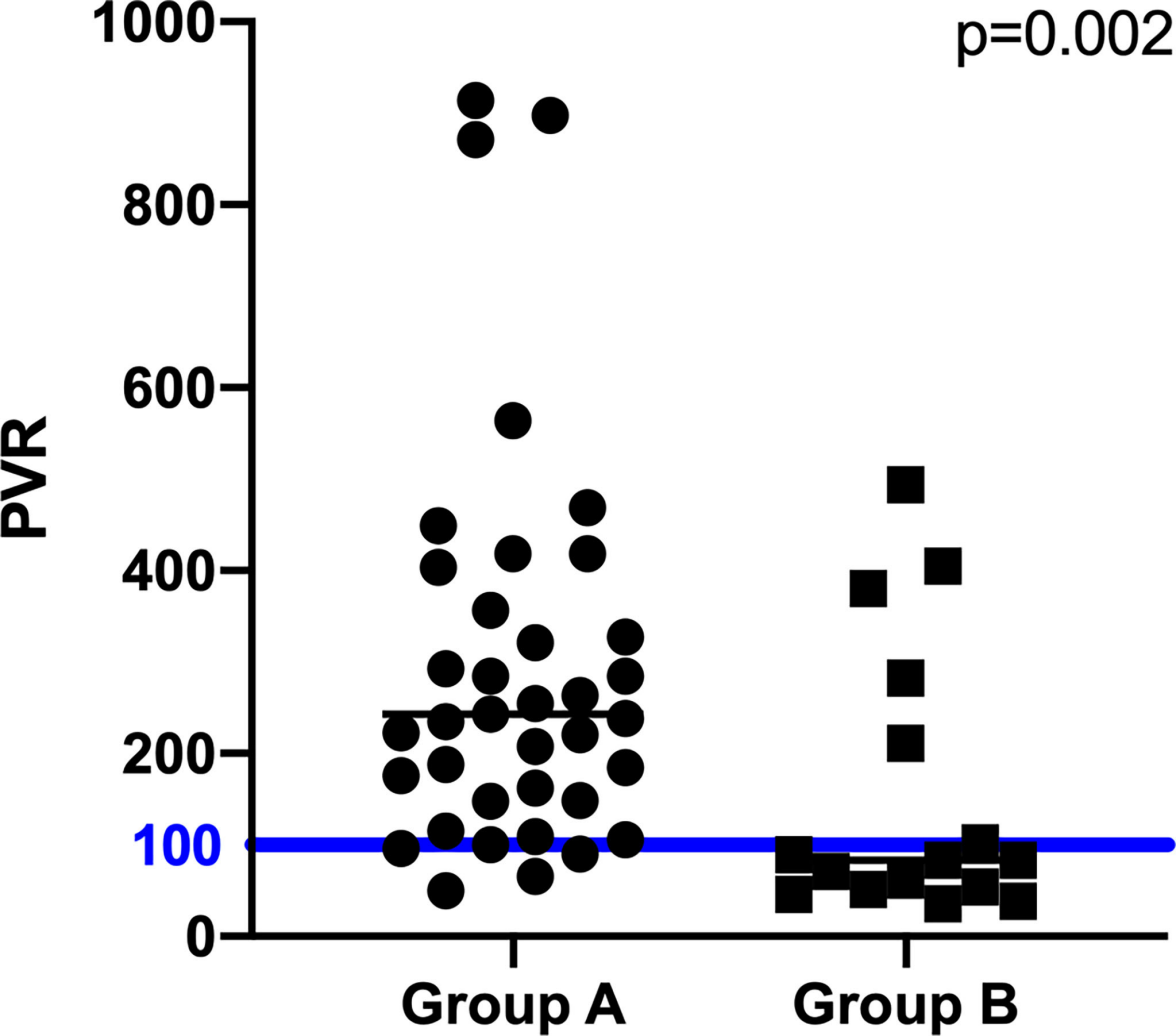
Differences of pre-operative PVR between group A, B. The blue line displays the PVR cutoff value of 100.

**Figure 3:**
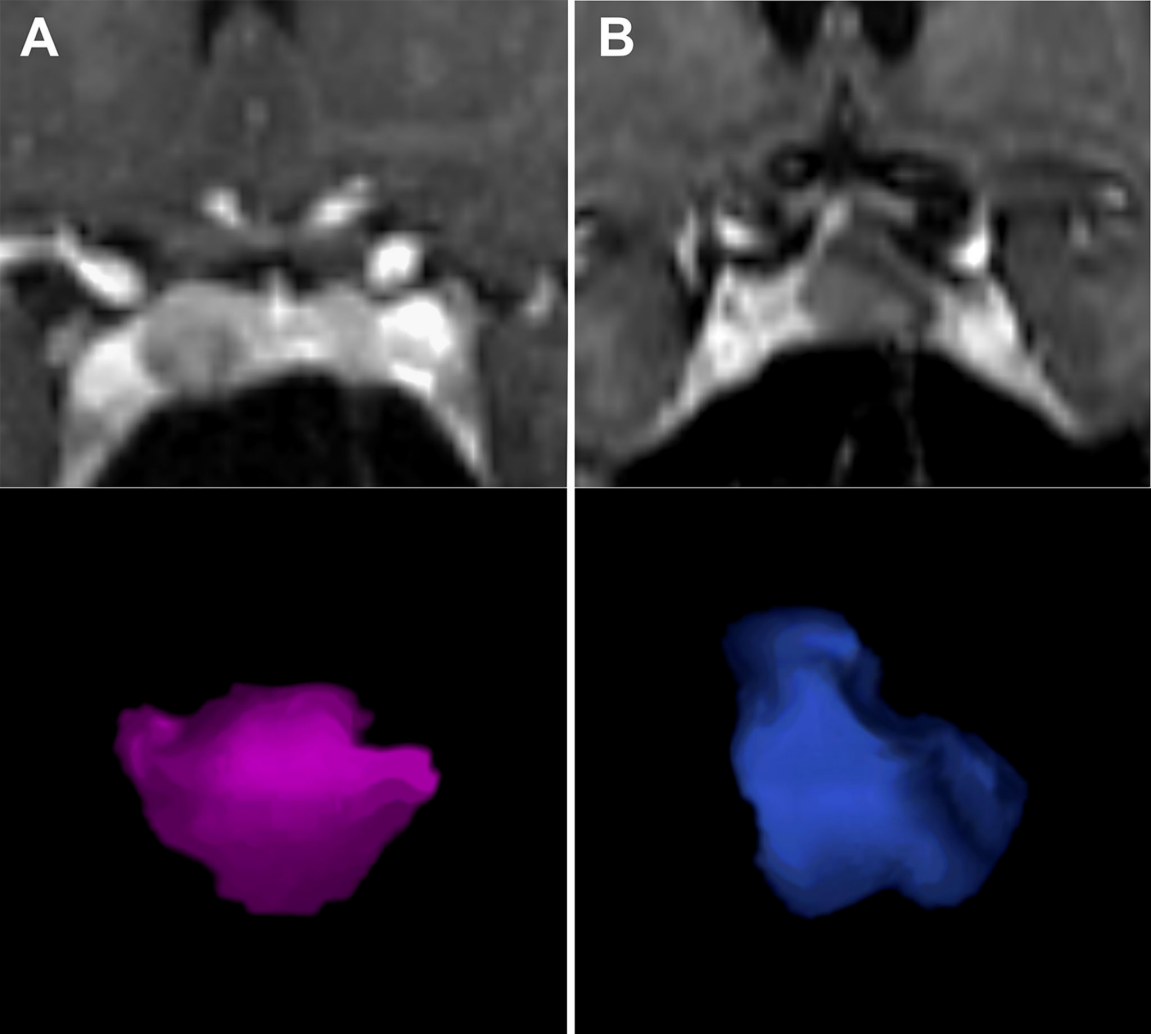
Application of PVR in two patients. **Figure 2** displays two patients with small sellar lesions with tumor volume of 0.5ccm^3^. Although pre-operative images represented similar findings, the PVR was significantly higher in **(A)** - a female patient with a PVR of 330 ng/mL/ccm^3^ and histologically confirmed prolactinoma. In comparison, **Figure 2B** shows a female patient with a PVR of 90 ng/mL/ccm^3^ and histologically confirmed Rathke’s cleft cyst. PVR, Prolactin-Volume-Ratio. **(A)** left side (MRI and pink rendered image); **(B)** right side (MRI and blue rendered image).

Subsequently, the Prolactin-Volume-Ratio (PVR) was tested for its predictive value to distinguish group A from group B. In group A, the median PVR was significantly higher (243 ng/mL/cm^3^, IQR 148 - 418) than in group B patients (83 ng/mL/cm^3^, IQR 52 - 247; p=0.002; **Figure 1C**). To identify the appropriate PVR that could predict prolactin-producing pituitary adenoma, a ROC curve analysis was performed. A threshold of PVR >100 showed an AUC of 0.77 (p=0.002), suggesting higher PVR as predictor for prolactin-producing lesions (**Figures 2**, **3**).

At this level, the predictive PVR model showed a sensitivity of 90% and a specificity of 38%.

Even in patients with prolactin-producing sellar lesions <1cm, the cutoff PVR > 100 showed a statistical tendency for predicting the pathology (p=0.059). Since the statistical analysis and results may be limited by the small number of patients in this category (n = 27), further studies with larger number of patients are of utmost interest.

Moreover, the extent of pituitary stalk deviation was shown to be of predictive value: Patients within group A showed a significantly lower degree of pituitary stalk deviation (6°, IQR 0 - 13) than patients within group B (14°, IQR 11 - 27; p=0.009; **Figure 1D**). Furthermore, in patients with stalk deviation (n=39), PVR >100 showed to be significantly predictive for the diagnosis of prolactin-producing sellar lesions (p=0.001).

Indeed, the presence of stalk compression did not demonstrate a predictive, diagnostic role (p=0.211).

### Follow-up

In a next step, we evaluated the postoperative change of the PRL levels. After surgical removal, a significant decrease in PRL levels could be observed in all patients.

At the time of last follow-up, a PRL of sex-corrected PRL normal range was reached in 97% in group A and 71% in group B. This resulted in a gross total resection of 95% in group A and 89% in group B.

### Laboratory and radiological differences between lactotroph and mammosomatotroph adenomas

To further differentiate lactotroph from mammosomatotroph adenomas, sub-analysis of group A patients was performed. Lactotroph adenomas were found in 31/39 (80%) patients, while 8/39 (20%) patients had mammosomatotroph adenomas. Between lactotroph and mammosomatotroph adenomas, no significant differences in preoperative PRL levels (p=0.251), lesion volume (p=0.223) and extent of pituitary stalk deviation (p=0.223) could be observed. Although non-statistically significant, the median PVR showed a tendency to differentiate lactotroph (264, IQR 176 - 449) from mammosomatotroph adenomas (167, IQR 106 - 254; p=0.065).

In patients with mammosomatotroph adenomas, median somatotropin level at diagnosis was 3.3 ng/ml (IQR 1.1 - 7.6) and median IGF-1 level was 460.5 ng/ml (IQR 292.0 - 627.5).

## Discussion

Preoperative knowledge if a small sellar lesion with elevated PRL levels is a prolactin-producing adenoma (prolactinoma) or another sort of sellar lesion is of utmost interest for patient counselling and therapeutic decisions (6, 9, 10).

Prolactinomas can be treated by medical treatment with DA, while other pathologies need a surgical resection to avoid further tumor growth or worsening of neurological symptoms, respectively being managed conservatively with only clinical and radiological follow-up (3, 6, 10). Up to 35% of pituitary adenomas are known to be nonfunctioning adenomas and were often found incidentally, allowing the conservative management of these patients (10).

To distinguish prolactinomas from other pituitary adenomas, several studies have investigated a variety of preoperative characteristics, such as serum prolactin levels or tumor volume (1). Historically, sellar lesions causing serum prolactin levels greater than 200 ng/mL were considered to be prolactinomas (6, 11, 12). However, recent studies have reported lower cutoff values, such as 39 or 56 ng/mL, for diagnosis of prolactinomas (5, 13, 14). Nevertheless, these prolactin cutoff values were mainly applied to differentiate prolactinomas from other pathologies in mixed cohorts, irrespectively of their tumor size (11). Consequently, we investigated various patients’ and tumor characteristics in a selected cohort of patients with small sellar lesions (≤15mm) to find appropriate preoperative diagnostic criteria.

We could show that patients with prolactin-producing pituitary adenomas (Group A) were significantly younger, had higher pre-operative prolactin levels, and smaller lesions with lower degree of pituitary stalk deviation.

Furthermore, the ratio of prolactin levels to tumor volume (PVR) was likewise shown to be predictive for the differentiation of prolactinomas from other types of sellar lesions causing hyperprolactinemia (5). However, Huang et al. solely investigated pituitary adenomas with a diameter of >10 mm (5). Since the clinical relevance for patients with microadenomas or other small sellar lesions was still missing, we applied the PVR to patients with small sellar lesions (≤ 15mm) and mild hyperprolactinemia (≤ 150 ng/mL).

Thereby we could also show that PVR is highly predictive for differentiating prolactin-producing lesions from non-prolactin-producing lesions.

Best to our knowledge, our study is the first study that investigates PVR in patients with small sellar lesions. Furthermore, ROC curve analysis (AUC of 0.77) revealed that pre-operative PVR >100 may be of prognostic value for predicting prolactin-producing pituitary adenomas.

In small sellar lesions with minor stalk deviation, precise preoperative diagnosis of the type of pathology may be impeded by the mild prolactin elevation caused either by prolactinoma and/or the stalk effect (4). In our study, the extent of pituitary stalk deviation was shown to be predictive for the diagnosis of prolactin-producing lesions. While patients with prolactin-producing lesions had higher prolactin levels with lower degree of stalk deviation, patients with non-prolactin-producing lesions had a higher degree of the stalk deviation, mimicking the mild prolactin elevation. This mild prolactin elevation in non-prolactin-producing lesions could be explained by the pituitary stalk effect, the most recognized hypothesis for hyperprolactinemia in nonfunctioning pituitary adenomas (10). Therefore, the extent of stalk deviation should also be taken into consideration to differentiate between prolactin-producing and non-prolactin-producing lesions.

### Limitations

The main limitations of our study are its retrospective design as well as the relative small number of patients included in this single center trial. Furthermore, there is a potential for a selection bias, due to a non-randomized treatment cohort.

However, this study can serve as an inducement to perform large prospective trials to verify the cuttoff PVR > 100 for small sellar lesions.

## Conclusion

In patients with small sellar lesions, Prolactin-Volume-Ratios >100 represents a possible predictive marker for the diagnosis of prolactin-producing pituitary adenomas.

## Data availability statement

The datasets presented in this article are not readily available because of the ethics committee. Requests to access the datasets should be directed to stefan.wolfsberger@medunigraz.at.

## Ethics statement

The studies involving human participants were reviewed and approved by the ethics committee of the Medical University of Vienna. Written informed consent for participation was not required for this study in accordance with the national legislation and the institutional requirements.

## Author contributions

AC, WM, SK, SW and AM contributed to data acquisition. AC, SW, and AM drafted the initial version of the manuscript, tables, and figures. AC and AM performed the statistical analyses. All authors were involved in data interpretation and editing of the manuscript. All authors have read and agreed to the published version of the manuscript.

## Conflict of interest

The authors declare that the research was conducted in the absence of any commercial or financial relationships that could be construed as a potential conflict of interest.

## Publisher’s note

All claims expressed in this article are solely those of the authors and do not necessarily represent those of their affiliated organizations, or those of the publisher, the editors and the reviewers. Any product that may be evaluated in this article, or claim that may be made by its manufacturer, is not guaranteed or endorsed by the publisher.
